# To vaccinate or to isolate? Establishing which intervention leads to measurable mortality reduction during the COVID-19 Delta wave in Poland

**DOI:** 10.3389/fpubh.2023.1221964

**Published:** 2023-09-07

**Authors:** Marcin Piotr Walkowiak, Dariusz Walkowiak, Jarosław Walkowiak

**Affiliations:** ^1^Department of Preventive Medicine, Poznan University of Medical Sciences, Poznań, Poland; ^2^Department of Organization and Management in Health Care, Poznan University of Medical Sciences, Poznań, Poland; ^3^Department of Pediatric Gastroenterology and Metabolic Diseases, Poznan University of Medical Sciences, Poznań, Poland

**Keywords:** COVID-19, SARS-CoV-2, vaccination, evidence-based policy, flattening the curve

## Abstract

**Background:**

During the Delta variant COVID-19 wave in Poland there were serious regional differences in vaccination rates and discrepancies in the enforcement of pandemic preventive measures, which allowed us to assess the relative effectiveness of the policies implemented.

**Methods:**

Creating a model that would predict mortality based on vaccination rates among the most vulnerable groups and the timing of the wave peak enabled us to calculate to what extent flattening the curve reduced mortality. Subsequently, a model was created to assess which preventive measures delayed the peak of infection waves. Combining those two models allowed us to estimate the relative effectiveness of those measures.

**Results:**

Flattening the infection curve worked: according to our model, each week of postponing the peak of the wave reduced excess deaths by 1.79%. Saving a single life during the Delta wave required one of the following: either the vaccination of 57 high-risk people, or 1,258 low-risk people to build herd immunity, or the isolation of 334 infected individuals for a cumulative period of 10.1 years, or finally quarantining 782 contacts for a cumulative period of 19.3 years.

**Conclusions:**

Except for the most disciplined societies, vaccination of high-risk individuals followed by vaccinating low-risk groups should have been the top priority instead of relying on isolation and quarantine measures which can incur disproportionately higher social costs. Our study demonstrates that even in a country with uniform policies, implementation outcomes varied, highlighting the importance of fine-tuning policies to regional specificity.

## Introduction

In early 2019, the World Health Organization (WHO) issued guidelines concerning flu pandemics. Even in the worst-case scenario of new variant with “no-specific vaccines,” “no pre-existing immunity in the human population” and the “extraordinary” severity leading to pandemic causing “potentially millions of deaths,” no quarantine was recommended, while those infected were advised to voluntarily self-isolate until they no longer had major symptoms. The most far-reaching recommendations included short school closures with the encouragement of remote working and modest international travel restrictions (1). Slightly adjusted guidelines for containing some extraordinary flu strains would seem a natural policy response as COVID-19 was causing mortality well within what was observed during the worst three flu outbreaks of the twentieth century and closer to the less wellknown ones of 1957 and 1968 (2). Prior studies based on the 2002–2004 SARS epidemic showed that the effectiveness of quarantine policies varied among countries and that they were important in containing the initial outbreak (3). In the case of influenza, quarantines were considered one of the least cost-effective interventions but a high level of compliance was considered unlikely (4). However, those calculations assumed having antivirals and vaccines providing modest cross-immunity, thus it was necessary to use what was available for this particular virus, and in this particular case, harsh non pharmaceuticals interventions (NPIs) were the only viable tool in the initial COVID-19 pandemic (5). Such a policy shift was augmented by the argument that the countries implementing restrictions were imitating those introduced in neighboring countries (6).

The implementation of these policies, along with emerging research studies (7), gradually contributed to the development of evidence-based strategies for managing the pandemic, particularly in terms of their cost-effectiveness (8). There is evidence indicating that a consistent and successful approach to virus containment can not only save lives but also support the economy (9). For instance, Japan achieved remarkable success in containing the virus, despite a slower vaccine rollout (10). However, these strategies were effective primarily in countries with high levels of social discipline or in geographically remote areas, offering limited guidance on how to proceed once initial containment efforts failed. While lockdowns were initially popular, they later became viewed as having disproportionate costs. Studies have also shown that their effectiveness varied across countries depending on the quality of governance (11) such as in Chile where their impact was only noticeable in higher-income areas in the country (12). Over time, the approach has shifted toward a more moderate stance, encouraging outdoor activities and emphasizing education and harm reduction over coercion (13).

Looking back at the COVID-19 pandemic, it is crucial to move away from relying solely on countries deemed exemplary (13) or models based on idealized human behavior. Instead, we should conduct a retrospective analysis of available data to identify the effectiveness of commonly adopted policies like vaccination, isolation, and quarantine, which were widely recognized within the global health community. This analysis will allow us to discern measures that have proven effective, even under less than ideal implementation. Recognizing the multifaceted challenges posed by both less-than-optimal governance structures, societal fatigue, and the highly dynamic situation with the virus undergoing evolution, it is of utmost importance to identify and prioritize policies that demonstrate resilience in the face of these obstacles. By drawing insights from these observations, we can better prepare ourselves for potential future pandemics.

## Methods

From the beginning of the pandemic to the end of October 2022, Poland had not reached the threshold of performing an average of one COVID-19 test per inhabitant, therefore the number of infection cases could not be used as a reliable indicator of the scale of the pandemic at a given moment. Using registered COVID-19 deaths was also unreliable, as prior studies showed that only ~2/3 of actual deaths were detected in the 2020/21 season (14). Therefore, we decided to analyse the effectiveness of applied methods indirectly by measuring excess deaths attributable to COVID-19 and the timing of detecting the wave peak which can be used as a metric of at least slowing down the virus spread.

### Outcome metric selection

Given the gradual spread of the Delta variant in Poland from as early as April 2021, the temporal dynamics of the autumn epidemiological situation were determined by ability to maintain low reproduction number. To fit our regression model, the calculation of COVID-19-related excess deaths was required for smaller regions. Weekly death data, represented in ISO weeks with 52 or 53 weeks per year from Monday to Sunday, were provided by Statistic Poland for 73 NUTS-3 level sub-regions. These data were then analyzed using hierarchical mixed Poisson linear regression to estimate the number of excess deaths associated with the virus. To address the extended period of elevated mortality, a mixed model was employed to eliminate excess values during baseline fitting. Furthermore, given the intended precision and resulting small sample size for regional data, a Poisson distribution was assumed and a hierarchical model was applied. Initially, the model was fitted to the whole country to generate the sinusoidal-like wave representing annual periodicity in the number of deaths. Subsequently, subregional models were given as input for the wave and dataset with already excluded periods of highly elevated mortality. Additionally, the fitting spanned the period 2002-W26 to 2022-W34 (format: yyyy-Www), weighted with age and sex composition as of the end of 2021 and where applicable, confidence intervals were approximated using the Monte Carlo method with the application of 50,000 simulated scenarios. The following regression model of fitting excess death was used:

For all of Poland:


$$\begin{array}{l} \ln(Y_{i})\;=\;a+b\left(T+\frac{t}{52.25}\right)+csin\left(T+\frac{t}{52.25}\right)\\ \;+\;dcos\left(\frac{2\pi t}{52.25}\right)+e+\epsilon \end{array}$$

For sub-regions:


$$\begin{array}{l} \ln(Y_{i})\;=\;a+b\left(T+\frac{t}{52.25}\right)\\ \;+\;c\left(c_{PL}\sin\left(\frac{2\pi t}{52.25}\right)+d_{PL}\cos\left(\frac{2\pi t}{52.25}\right)\right)+e+\epsilon \end{array}$$

Where,

Y_i_—the predicted number of weekly deaths,

a—base number of weekly deaths,

b—annual change in the number of weekly deaths,

T—year,

t—ISO week of the year,

c, d—season-dependent component of weekly deaths, created by superimposed sine and cosine function,

c_PL_, d_PL_—season-dependent component of weekly deaths fitted for Poland,

e—the binary variable that switches from 0 to 1 on 2020-W01 which adjusts for elevated mortality during pandemic not caused directly by the virus, ε–error of prediction.

A Poisson regression model was first fitted to the dataset for Poland. Subsequently, standardized residuals were calculated to weight the model in the next step. The standardized residuals that deviated upwards by 2 standard deviations had their weight set to 0. For improved precision, this process was iterated with weights taken from the weighted model. The subregional model used a seasonability variable derived from the country-level model. Additionally, input mortality data for this model was filtered so that the periods in which the country-level model had excess mortality exceeding 3 standard deviations were not included. The whole iterative process was repeated for sub-regions, as well as filtering out observations diverging upwards by more than 2 standard deviations from their respective models. Delta wave COVID-19 deaths were excess deaths that occurred from 2021-W36 to 2022-W02 (in yyyy-mm-dd format: 2021-09-06 to 2022-01-16) or to adjust for reporting delay, were officially reported as caused by COVID-19 between 2021-W37 to 2022-W03. Filter calculating peak of detected Delta wave infection was applied from 2021-10-05 to 2022-01-06.

### Input variable selection

Some theoretically available and seemingly suitable metrics were considered, although ultimately they were not used. The number of tests was inflated and the positivity rate deflated in cities with more hospitals where every patient was tested on admission. The relative number of cases, contact quarantines or the number of tests requested by GPs were also not used, as they were encompassing both the effectiveness of detection and local severity of the pandemic. The following variables were selected to indirectly gauge the pandemic response:

— Vaccination rate 70 and abov e/vaccination rate—respectively percentage of people 70 years and more and percentage of the general population who received at least a single dose of the COVID-19 vaccine as of 2021-11-28 to measure direct protection and herd immunity attributed to vaccination around the Delta wave peak.
— Detection ratio—the percentage of positive cases among vaccinated lower-risk groups (age 20–59, data collected in 10-year intervals) divided by the percentage of cases among vaccinated high-risk groups (age >60). This not only allows for the comparison of regions with different vaccination rates but takes into account the fact that the low-risk vaccinated people had most leeway in their behavior.
— Quarantines per case—the number of people quarantined (excluding border quarantine) in relation to the number of positive cases as a measure of the compliance and effectiveness of tracing contacts.
— Delay of cases peak/delay of deaths peak—the time difference between a local wave peak and the country average used to measure the slowing down of the spread. Gaussian filters were applied to establish the peak day of detection and peak week of excess deaths. In the case of infection, there was an additional problem of a weekly cycle caused by GPs' work schedules, with few infections detected at the weekend and disproportionately many on Mondays. Thus, for the detection of infection cases, a window of 21 days with a standard deviation of 0.3 per day was selected, since this allowed all days of the week to be almost equally weighted. Gaussian filter windows of 5 and 6 weeks with one standard deviation per week were applied to calculate the peak of weekly deaths.

### Data

The data on vaccination rates, sourced from the government program 'Ranking gmin' (“Municipalities ranking”) (15), aimed to incentivize higher vaccination rates through peer pressure and rewards for the top-performing municipalities. The cases detected between 2021-09-01 and 2021-12-31 were obtained from government website “Otwarte dane” (“Open data”) (16). To account for the lag between infection and death, the mortality was calculated for time frame delayed by approximately two weeks. The number of quarantines was obtained by virtue of the law on access to public information from the Ministry of Health.

### Models

In the first regression model analyzing was Poland divided in to 380 counties (“powiat”), the measured metric was the day of the peak wave based on officially detected cases. Since those regions were small, the model findings were also tested in a spatial regression model, incorporating the data from the six closest regions. The second model used data from Poland divided into 73 sub-regions (NUTS-3) to test to what extent the death rate among the 70+ population could be explained by their vaccination rate and the effectiveness of containing the pandemic measured by the delay of the peak of excess deaths, and whether the above-mentioned death rate could be explained by vaccination, case detection and contract tracing.

## Results

**Figure 1:**
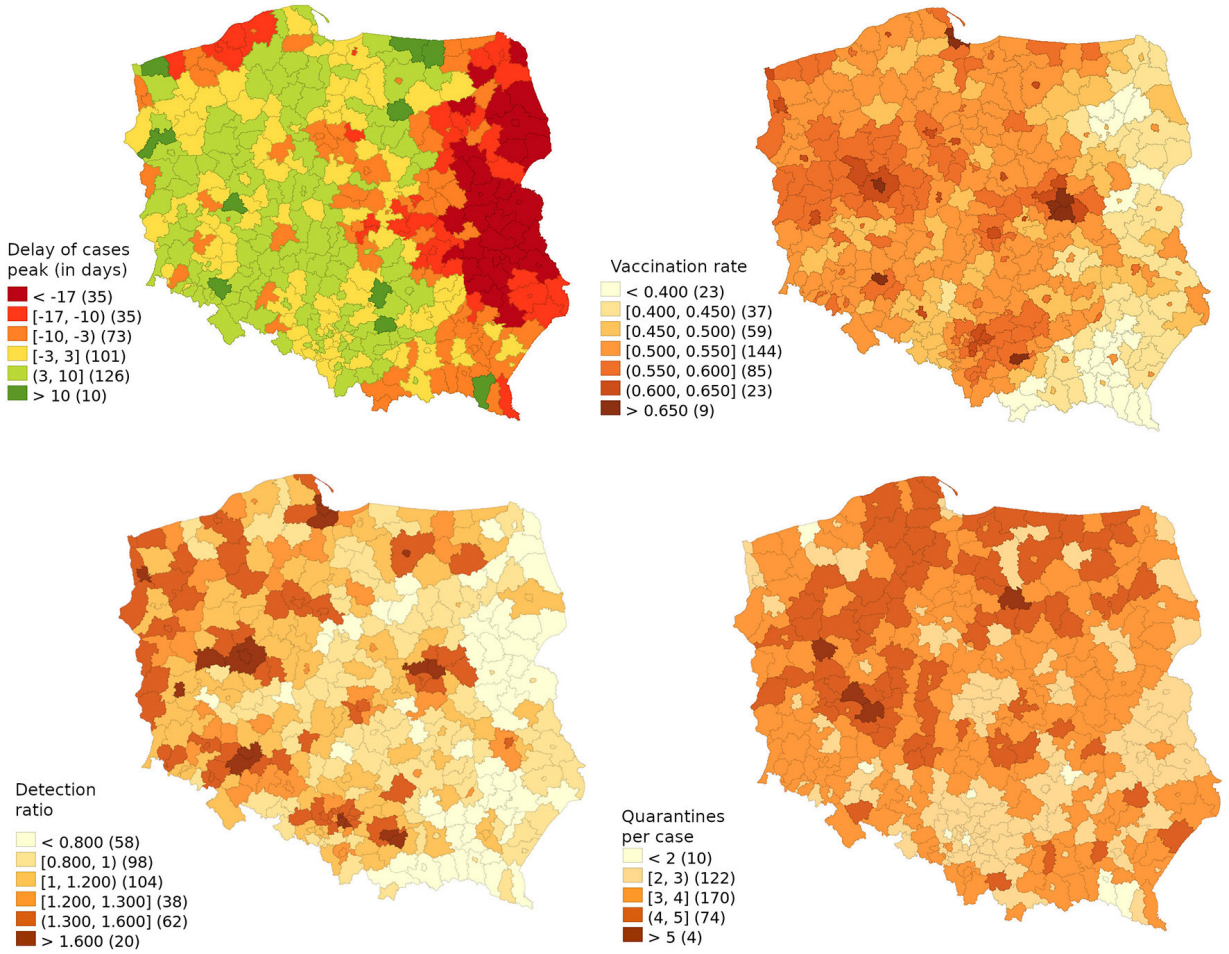
The delay of cases peak (in days) in relation to the country average with explanatory variables (vaccination rate, detection ratio and number of quarantines per case).

While the number of cases was steadily growing from July 2021, the Delta wave detection peak occurred on 2021-11-30 and the standard deviation was 10.25 days, as shown in Figure 1 and explained in the models in Table 1. The apparent imbalance on the map showing most of the country performing worse than the average was caused by big cities performing relatively well, while the peak come much earlier in less populated eastern regions.

**Table 1:** Regression models explaining the factors predicting the peak Delta wave.

	**Linear regression model**	**Spatial linear regression model**
Const	−40.938 (4.601)^***^	−12.793 (3.177)^***^
Spatial lag		0.813 (0.034) ^***^
Detection ratio	4.885 (2.463)^*^	
Quarantines per case	3.028 (0.652)^***^	1.166 (0.418)^**^
Vaccination rate	49.664 (9.957)^***^	16.439 (4.816)^***^
R^2^/Adjusted R^2^	19.6%/19.0%	68.8%/66.1%

^*^p < 0.05, ^**^p < 0.01, ^***^p < 0.001, standard deviation in bracket.

The detection ratio was highly correlated with vaccination rates, thus it lost its statistical significance in the spatial regression model. The spatial lag variable explained 81.3% of the explained variability. However, variables responsible for the number of quarantined contacts and vaccination share remained statistically significant, thus even though the infection spread throughout the country, the model showed that local factors maintained relevance.

Each vaccinated percentage point of the population delayed the peak by 0.500 days, thus a vaccination rate of 54.76% could lead to a delay of 27.2 days. This means that to delay the wave peak by one day, one would have needed to vaccinate an additional 2.01% of the population. The relative ratio of detection among vaccinated 20-59-year-olds in relation to those 60 and over should represent 6.23 days of delay as there was an overall 28% higher share of positive cases among the working-age population than among the retired. It is highly unlikely that the key transmission vector was working-age vaccinated or that the denominator fully reflected the infection rate among elders as the COVID-19 death rate among them was underestimated. Thus, using this variable as a proxy of the general quality of detection, for additional delay of the peak wave by one day one would need to detect 16.0% more cases, that is, to diagnose and isolate an additional 0.53% of the population. The 3.03 quarantines per detected case were responsible for delaying the wave peak by 9.19 days, thus to delay the peak by one day, additional contacts consisting of 1.08% of the population should have been traced and forced to stay at home.

**Figure 2:**
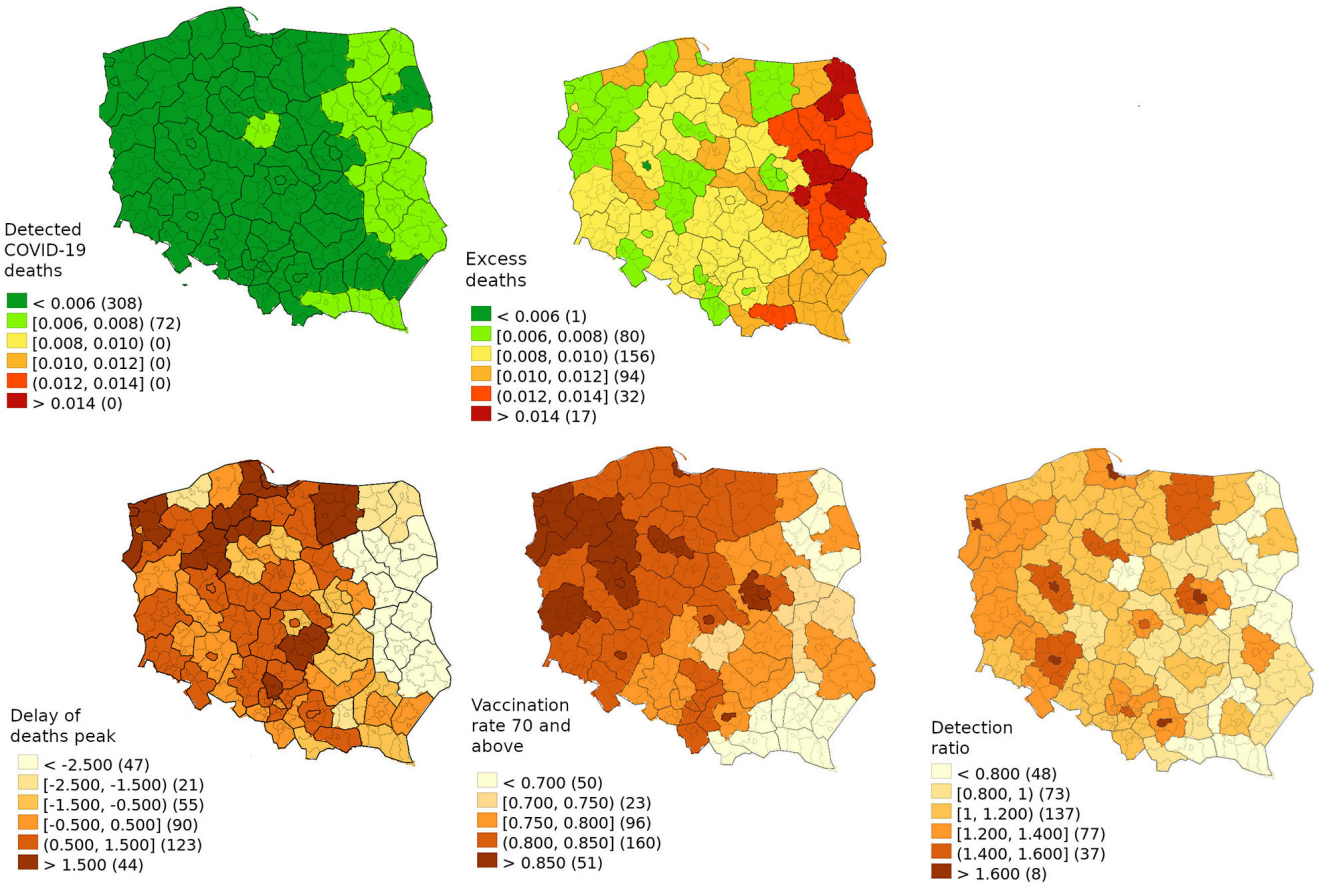
Delta wave death of people of 70 and above from the excess death model and official data with explanatory variables (delay of deaths peak, vaccination rate of 70 and above, detection ratio).

In Figure 2, Poland is subdivided into 73 sub-regions to compare the risk of COVID-19 death among those 70 years and above based on the excess death model, official data and relevant explanatory variables, while Table 2 presents the models explaining the excess death in this age group. Based on superior adjusted R^2^ 68.3 vs. 54.9% peak wave delay has a higher predictive value than any other marker of intervention. Differences in the compliance and enforcement of the quarantine policy among regions were not sufficiently large to generate a statistically significant variable on its own.

**Table 2:** Regression models predicting the risk of death among 70 and more during the Delta wave.

	**Model including delay of the peak**	**Model excluding delay of the peak**
Const	0.0220 (0.00219)^***^	0.0227 (0.00303) ^***^
Delay of deaths peak	−0.000662 (0.000101)^***^	
Vaccination rate 70 and above	−0.0177 (0.00276)^***^	−0.0144 (0.00474)^**^
Detection ratio		−0.00294 (0.000970) ^**^
R^2^/Adjusted R^2^	69.2%/68.3%	56.2%/54.9%

^*^p < 0.05, ^**^p < 0.01, ^***^p < 0.001, standard deviation in bracket.

Considering that the model with peak delay without any interventions, the virus would kill 101,459 high-risk individuals so vaccinating those at high-risk saved 65,880 lives. Based on the first model, the interventions delayed the peak by 6.1 weeks, thereby saving 18,603 lives. As 70.5% of excess deaths occurred in those 70 and above, delaying the wave by one week is estimated to save 939 individuals or reduce excess death by 1.79% (1.26–2.33%).

Combing factors explaining the delay from Model 1 with the first variant of Model 2 which shows the relationship between the delay and lives saved allows us to calculate the number required to save a single life. The most effective way was simply to vaccinate 57 (43–81) people aged 70 or older. Furthermore, saving one life through achieving partial herd immunity required vaccinating 1,258 (687–2,276) low-risk individuals. Alternatively, one could detect 334 cases (122–38,148) and with 11 days of isolation keep patients isolated for a total of 10.1 years (3.7–1,150). Another option is tracing 782 (361–1,278) contacts and assuming a delay leading to 9 days of isolation, this would require forcing them to stay at home for 19.3 (8.9–31.5) years. For comparison, the average person dying of COVID-19 was 76.5 years in that period while before the pandemic, a similarly aged individual would be expected to live for 10.7 years (17). The above-mentioned calculation includes only interventions during the Delta wave and taking into account border quarantines or the continuation of this policy during the highly infectious Omicron wave would further reduce the effectiveness.

## Discussion

The applied models allowed us to assess the relative effectiveness of different public health interventions in saving lives during the pandemic. The most effective intervention to reduce mortality was the vaccination of high-risk individuals. While even countries with higher vaccination rates failed to achieve herd immunity, the second best policy remained the vaccination of low-risk individuals, as it produced clear gain among the more vulnerable. Furthermore, despite the less than ideal execution of isolation and quarantine measures (18), they played a role in preserving lives. The key beneficiary of those policies were those at high-risk who despite numerous campaigns refused to get vaccinated, while the cost of the policy was felt the most by society in general, especially children as the low-risk segment of society.

Our findings are consistent with the susceptible-exposed-infectious-recovered (SEIR) model of Pei et al. (19) showing on international sample that an increased COVID-19 vaccination rate slowly shifted the infection rate toward a much lower equilibrium. Our results are also in line with those of Ge et al. (20) with respect to the changing relationship between the role of NPIs and vaccines in containing the virus in Europe. While our model somewhat favors vaccination more than theirs, we compare its relative effectiveness vis-à-vis isolation and quarantine, while they compare it with NPIs in general, thus such a discrepancy is unsurprising. However, as they aimed to eradicate the virus, they reach a diametrically opposite conclusion policy-wise, as they recommended Europe in general to ramp up restrictions. Tang et al. (21) took into account game theory to explain behavior change, showing that in Western countries, in contrast to East Asia, a combination of pandemic fatigue and a rational decrease in voluntary social distancing was evident after the vaccination rollout. While this was interpreted by Tang et al. as evidence in favor of maintaining restrictive NPIs, this could also be seen as an argument supporting the position that non-Asian countries would have problems maintaining those restrictions and should concentrate instead on harm reduction policies and an exit strategy. Lin et al. (22) recommended continuation of isolation and quarantine well into the vaccine roll-out until herd immunity was achieved and even in their best-case scenario, an aggressive policy of isolation and quarantine was expected to reduce the number of cases by <20%, while the main gain was averting healthcare system overload. Their models also show vaccination is superior to isolation as a containment tool. Shattock et al. (23) found that in the case of a swift total lift of restrictions combined with a rapid vaccination campaign, there would still be problems containing the virus spread even though the overall number of deaths would be reduced. Contrary to the applied policies, these studies implicitly suggested the lifting of restrictions in the late vaccine rollout and based on this study, their conclusion held even when the vaccine began losing efficiency. Sonabend et al. suggested that lifting interventions must be balanced carefully and cautiously with the vaccine roll-out. In the presence of a new, highly transmissible variant, vaccination alone might not be enough to control COVID-19 (24). We intentionally concentrated on articles which were calibrated on data for an at least partially vaccinated population, as subsequent studies found that early COVID-19 pandemic prognostic models, when retrospectively analyzed, tended to have serious deficiencies. These deficiencies included overestimating the number of hospitalisations and deaths (25), as well as underestimating the impact of voluntary behavioral changes if the number of cases increased (26), or taking mutually exclusive assumptions concerning the reproduction number (27).

Policy-wise, instead of one-size-fits-all recommendations, a subtler approach is advisable. An additional problem is the issue of low-quality research into the effects of individual policies implemented in the fight against the pandemic (28, 29). This may also be the reason for not looking for individual and more nuanced solutions. Clearly, there were countries that thanks to their discipline and remoteness were able to contain the virus well. In the case of Japan, it was even possible to apply the opposite policy of containing infections while unhurriedly starting vaccination (10). A similar containment policy also proved effective during the first wave of the pandemic in South Korea (30), Taiwan (31) and Germany (32). However, when the WHO assessed the actual COVID-19 mortality rates, these countries stood out as outliers and it was suggested that the effectiveness of specific policies should not be solely evaluated based on this type of rankings, as other factors including compliance and local culture played a significant role in their outcomes (33). We would like to suggest going one step further and considering that not only did the overall effectiveness of the same preventive measures vary based on the local context, but there is also no reason to assume that their relative effectiveness or intrusiveness remained constant. As Redlin noted, “more prosperous countries implemented milder restrictions but responded more quickly, while poorer countries introduced more stringent measures but had a longer response time” (34). Assuming that there was political will for intrusive policies, the first obstacle was overcoming vaccine hesitancy in high-risk individuals. While hold-outs in this group turned out to be the most resistant to pressure, there are cases in Central and Eastern Europe of applying a combination of stimuli, both negative (COVID-19 passport) and positive (cash handouts) to achieve modest successes (35). However, it should be noted that the impact of such policies in countries with different levels of vaccination may not be the same (36). Additionally, as mass vaccinations striving for herd immunity worked more effectively than isolation and quarantine, the COVID-19 passport may be more palatable for low-risk groups. Attention was also drawn by researchers to the use of “package” lockdown policies by various countries, and the consequences of such solutions, for example from the point of view of equity harms of COVID-19 policy interventions (34) but the consequence of such “packages” is also a lack of prior analysis of what might work. Of course, in this situation, the issue of costs incurred also disappeared in terms of the economic costs incurred by individual states (37–39) but also of costs incurred by citizens (31, 40, 41). As Joffe wrote in the case of lockdowns, “It is past time to take an effortful pause, calibrate our response to the true risk, make rational cost-benefit analyses of the trade-offs, and end the lockdown groupthink” (6).

Nonetheless, some of the suggested measures bear a cost that cannot be neglected. In many countries national and local laws during the COVID-19 pandemic led to seriously compromising democracy and human rights (42, 43). In line with these concerns, the Polish government attempted to pass a special blanket provision exempting authorities from criminal liability for violating any laws if their intention was to combat COVID-19 (44). Furthermore, as the official report by the Supreme Audit Office stated, the government's actions, including arranging presidential mail-in voting without a legal basis, incorrect involvement of state institutions, violations of budget spending laws, and breaches of data protection laws, contributed to the cancellation of the elections (45). Additionally, the government passed a retrospective law granting immunity from prosecution to involved individuals (46). These actions highlight the delicate balance between public health measures and upholding democratic values. Moreover, while isolation and quarantine proved effective in controlling the spread of the virus, it is important to acknowledge the high psychological cost they impose on individuals (47, 48). Pandemic restrictions lead to decrease of avaibility of other medical services (49). The economic costs were also enormous including the increased unemployment rate, broken delivery chains, and company bankruptcies (50). As studies have shown that the absence of imposed NPIs is likely to be partially offset by people being more cautious, possibly a more benign system encouraging people to get tested and reveal contacts with no fear of state-imposed sanctions could have been somewhat less effective but at a dramatically lower social cost.

While the issues of democracy are the subject of academic or political discussions, scientific standards, including the ways to fight the pandemic, must result from scientific premises. The implementation of public policies does not take place in a vacuum, with both the local context and the need to achieve country-specific goals considered. Moreover, nothing can justify a policy that is only implemented in a certain way because it has always been implemented that way, or because neighboring countries do so.

In the scientific debate, there are also views that perhaps “policy-making during the COVID-19 pandemic has been biomedicinecentric in that its evidential basis marginalized input from non-biomedical disciplines” (51). In particular, effective, clear and transparent public communication appears mandatory for successful contagion containment policies (52). Regardless of the adopted policy, its success will also depend on the determination of the state to implement it, as well as how citizens follow the recommendations. The government may mandate the wearing of masks indoors but enforcing the restrictions is another matter. From this point of view, many works on policies to counteract the pandemic are devoid of deeper meaning because they assume that the policy announced equals the policy implemented, so the premise often appears counterfactual. Furthermore, the longer various Covid restrictions last, the more often they tend to be treated with indulgence and even boycotted.

The very wide confidence interval for the effectiveness of isolation is due to the p-value of the relevant variable in the model being slightly below 0.05, while the p-values of other variables were well below 0.01 or 0.001. In practice, the policies of stopping virus spread by forcing potentially infectious citizens to stay at home should have highly interrelated effectiveness, explainable by the actual viral load and the reduction of contacts. Consequently, while this cannot be calculated directly from our model, the effectiveness of isolation is highly unlikely to assume extreme values permitted by the confidence interval, as these would be difficult to reconcile with the effectiveness of quarantine.

The relationship between the number of years of life saved in relation to the number of years of life whose quality must have been lowered because of compulsory home confinement could be a reasonable trade-off, except for the fact that compliance with different policies was generally mutually highly correlated, and thus the model was most likely attributing gains from other interventions, such as wearing masks, toward the measured variables. However, there is a more fundamental issue, without the benefit of hindsight, one has to answer whether the sudden appearance of a highly infectious but milder variant could have been expected. If one considers this as a chance that should not be taken into account when shaping a policy, then policies stopping the spread, unless they eradicate the virus, would be less effective in the long run to prevent deaths. Alternatively one could consider the rise of a less virulent but more infectious variant as a highly likely scenario favored by evolution. In that case, buying time would be a highly rational policy but one should be expected to drop this policy as soon as the awaited milder variant is detected. This would mean that the continuation of this policy into the Omicron wave was undesired policy inertia of fighting the Delta variant, decreasing its overall effectiveness by increasing the number of isolation cases by 144.5% and quarantines by 78.8%.

The theoretically unfavorable cost-benefit relationship of these measures cannot be solely attributed to Poland, as the overall pandemic mortality in the country was comparable to that of other post-communist nations. Moreover, Poland's performance was even more successful when compared to certain European Union members, such as Romania or Bulgaria, who implemented similar approaches. The strategies' limited effectiveness does not solely stem from weak state institutions. Official statistics in Poland indicate that about half of the deaths occurred among unvaccinated individuals in high-risk categories. Improving the overall implementation of pandemic prevention measures would have raised vaccination rates in these vulnerable groups, reducing the potential benefits of curbing the virus's spread.

Additionally, the continuation of those policies unchanged during the Omicron wave was widely adopted as the mainstream approach. The ECDC recommended on 2022-01-07 maintaining the full duration of isolation and quarantine measures as the scientifically supported approach, while acknowledging the limited possibility of slightly reducing the duration only in exceptional cases of high or extreme pressure on the healthcare system (53). Thus, from the institutional perspective, dropping those policies at that moment was unlikely, therefore it is highly likely that the social cost incurred in this period should be considered as a practically unavoidable cost of applying those policies during the Delta wave. Moreover, stronger state institutions that would professionally implement those harsh policies could have turned into a liability, since a year after the peak of the Delta wave, the Koch Institute in Germany was persistently recommending compulsory isolation of COVID-19-positive individuals (54), thus reducing the overall effectiveness of the initially adopted policies.

### Limitations

When considering the official estimation of the number of years that a person dying of COVID-19 would otherwise live, it is important to acknowledge that this estimation should be treated as a rough approximation. It does not take into consideration factors such as the higher prevalence of pre-existing conditions among those who died or the potential long-term health implications caused by the virus among those who survived. The strong correlation among the explanatory variables indicates the presence of an underlying factor that influenced the level of commitment exhibited by the local population in fighting the pandemic. The observed variables, which showed a clear correlation, could be seen as manifestations of this underlying factor rather than independent factors themselves. On the other hand, there were also studies implying clearly heterogenic approach within population, as there was both detected a significant, affluent, reasonably educated cluster which was strongly against restrictions and was consider government of fearmongering, while simultaneously their socio-economical features otherwise were predictor of high vaccine acceptance (55).

The observed variables, which showed a clear correlation, could be seen as manifestations of this underlying factor rather than independent factors themselves. On the other hand, other studies have suggested a clear heterogeneity acceptance of different pandemic prevention measures. A cluster analysis identified a group that displayed the strongest opposition to restrictions, questioning the accuracy of reported death statistics and perceiving the official messaging as fearmongering (55). This group had socio-economic characteristics resembling a middle-class population residing in sub-urban areas, which would otherwise predict above-average vaccine acceptance.

Additionally, the model assumes that the observed proxy regional variables remained effectively constant or evolved in the same direction within the analyzed time frame. However, based on studies observing long-term COVID-19 vaccination trends in Poland, it appears that the analyzed regions were likely undergoing subtle divergence (56, 57). Furthermore, it should be noted that due to the limited number of observations, only linear models could be fitted. While these models provide insights into marginal trade-offs with modest policy adjustments, it is suspected that the actual relationships could be non-linear. Therefore, extrapolation to entirely different policies should be approached with additional caution. Modeling of excess mortality is based on methodology used by EuroMoMo (58), thought with additional assumption of chierarchical model. This approach is generally suitable for detecting sudden surge in mortality, though it may underdetect in case of long periods of slightly elevated mortality.

## Data availability statement

Publicly available datasets were analyzed in this study. This data can be found here: https://bdl.stat.gov.pl/bdl/start; https://web.archive.org/web/20211214141631/https://www.gov.pl/web/szczepienia-gmin/sprawdz-poziom-wyszczepienia-mieszkancow-gmin; https://dane.gov.pl/pl/dataset/2582,statystyki-zakazen-i-zgonow-z-powodu-covid-19-z-uw.

## Author contributions

MW, DW, and JW conceived and designed the study, interpreted the data and drafted the figures, developed the analytical strategy, and had full access to all data used in this study. MW and DW obtained and managed the data. MW conducted analysis and wrote the first draft of the manuscript. All authors were responsible for submitting the article for publication and provided input to finalize the paper.
