# Supplementary material for: To vaccinate or to isolate? Establishing which intervention leads to measurable mortality reduction during the COVID-19 Delta wave in Poland
Source: Front Public Health. 2023 Sep 7;11:1221964. doi: 10.3389/fpubh.2023.1221964 (PMC10513426; doi:10.3389/fpubh.2023.1221964)
Supplement: Supplementary file 1 [file Data_Sheet_1.docx]

Suplementary materials


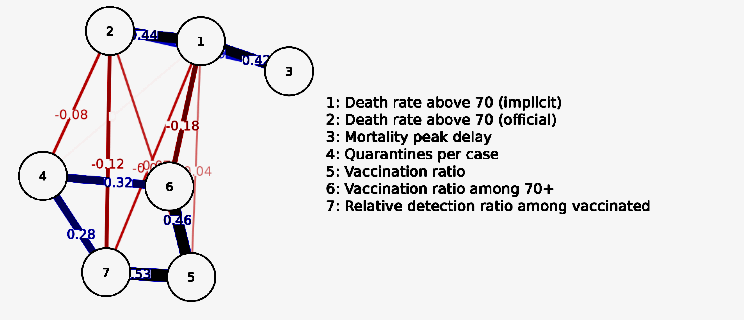


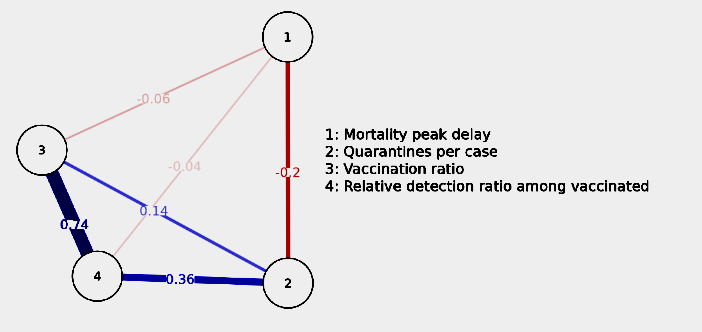


Figure S1: Network analysis of relationship between variables


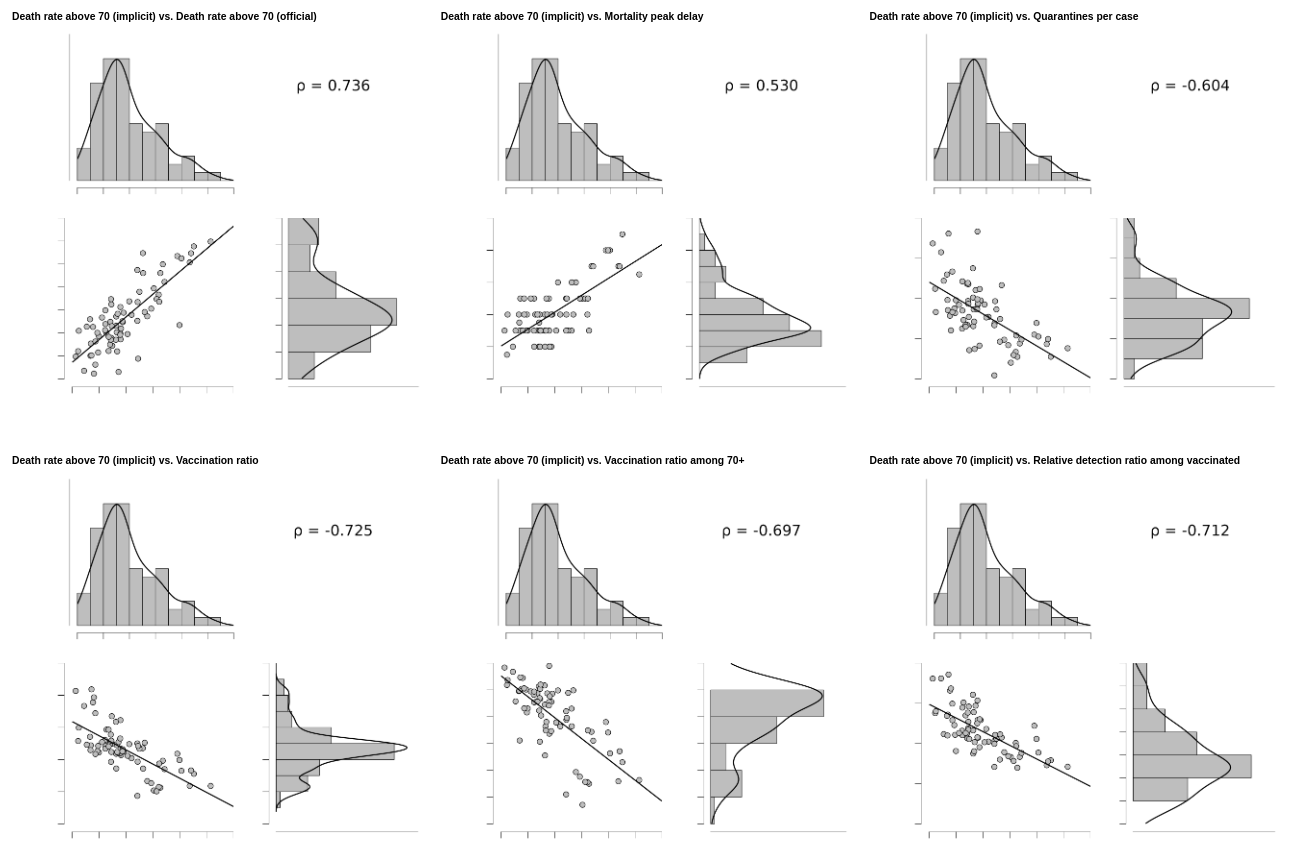


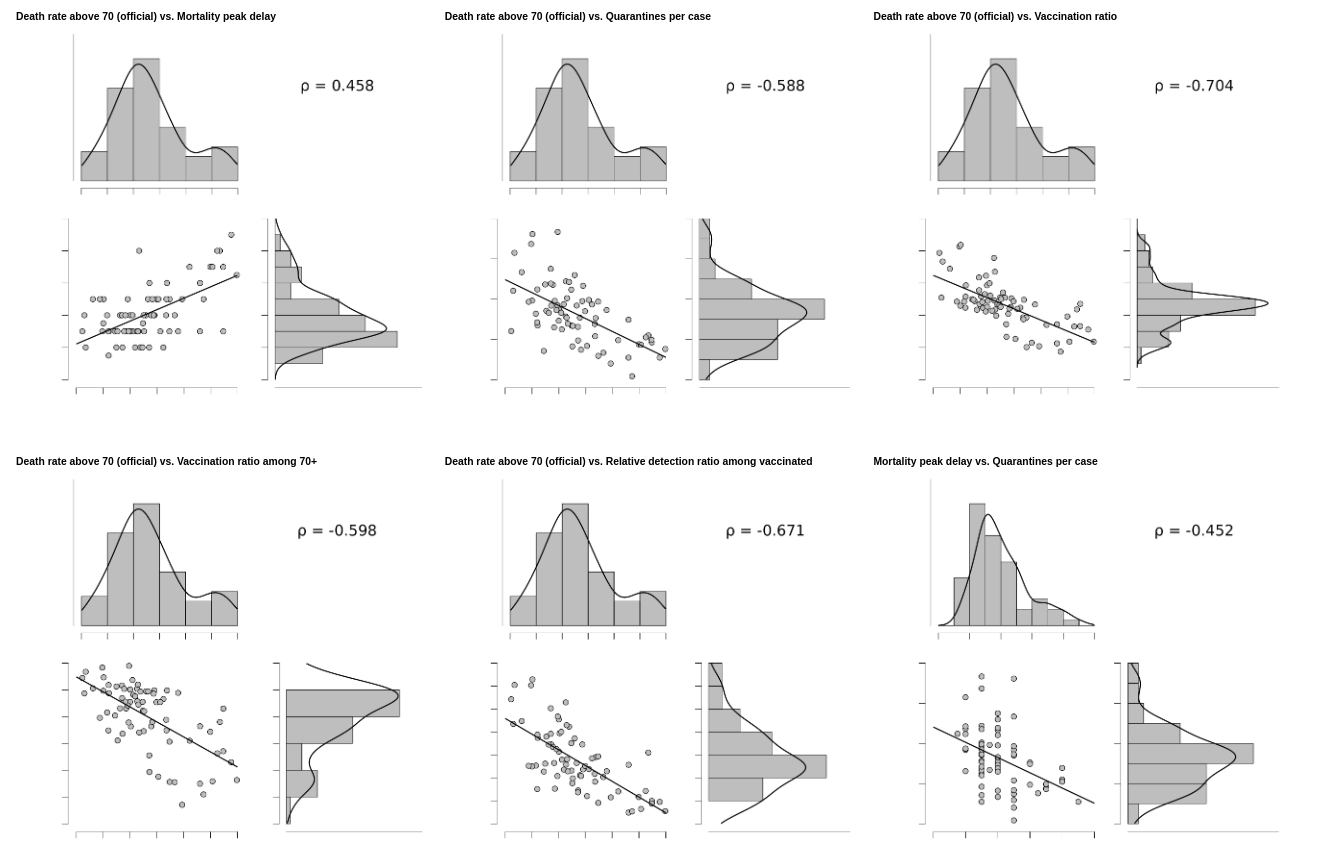
Figure S2.1: Correlations between variables part 1


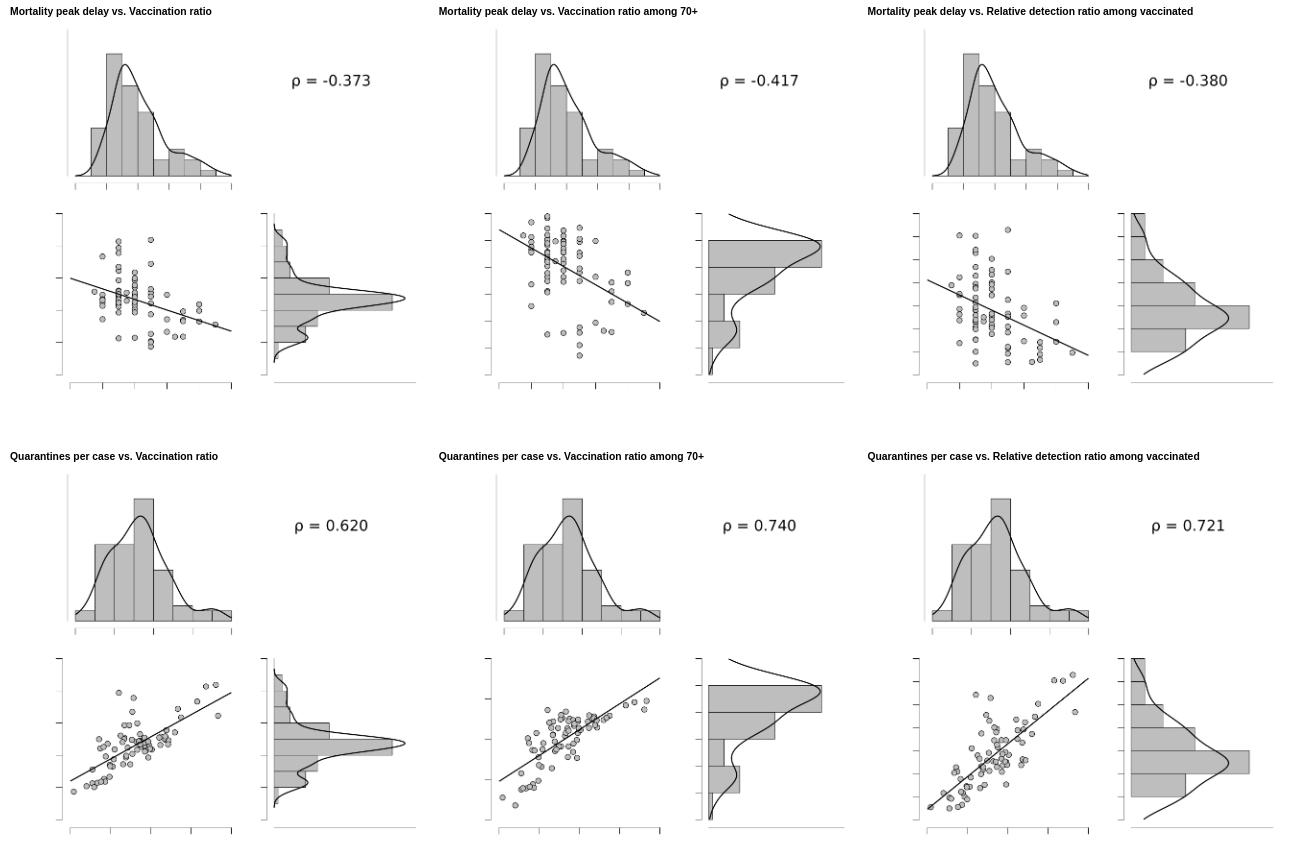


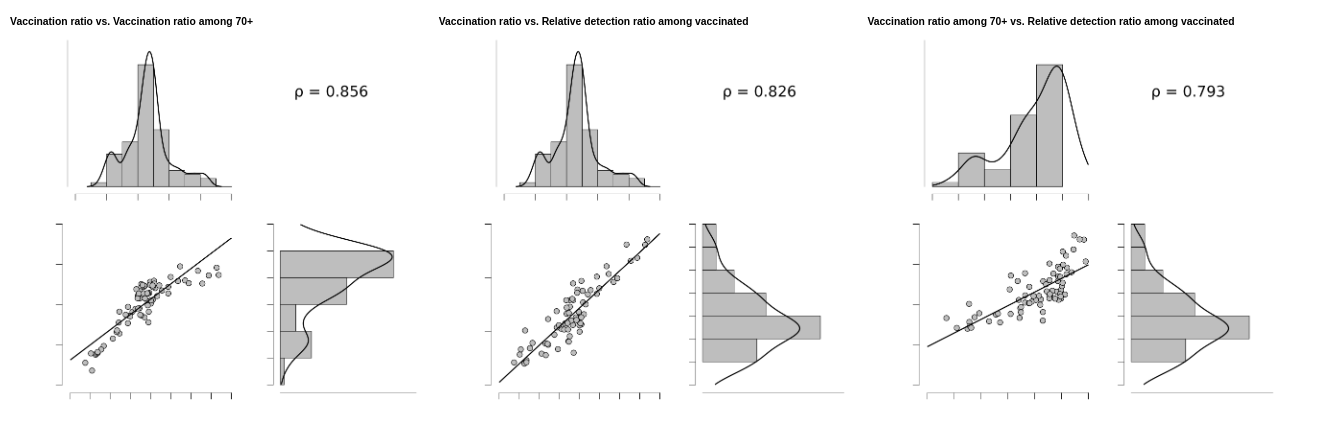


Figure S2.2: Correlations between variables part 2

| Mean | -2.1368 |
| --- | --- |
| Median | 1.0000 |
| Minimum | -41.000 |
| Maximum | 18.000 |
| Standard deviation | 10.253 |
| C.V. | 4.7983 |
| Skewness | -1.0756 |
| Ex. kurtosis | 0.87039 |
| 5% percentile | -25.000 |
| 95% percentile | 9.0000 |
| Interquartile range | 14.000 |
| Missing obs. | 0 |

Table S1: Summary statistics, using the observations 1 – 380 for the variable '4thWaveShift' (380 valid observations), staiting daily shift of infection peak in relation to peak for the whole country.
